# Cutaneous Basal Cell Carcinoma with Bone Metastases: An Orthopaedic Case Report

**DOI:** 10.1155/2019/1628980

**Published:** 2019-06-23

**Authors:** Brianna R. Fram, John Strony, Geetha Jagannathan, Scot A. Brown

**Affiliations:** ^1^Department of Orthopaedic Surgery, Thomas Jefferson University Hospital, Philadelphia, PA, USA; ^2^Rothman Orthopaedic Institute, Philadelphia, PA, USA; ^3^Department of Pathology, Anatomy, and Cell Biology, Thomas Jefferson University Hospital, Philadelphia, PA, USA

## Abstract

Basal cell carcinoma (BCC) is the most common skin cancer, and its incidence is increasing. Though metastatic BCC (mBCC) is uncommon, the literature demonstrates a 0.0028%-0.55% rate of metastasis. We report on a patient treated at our institution who was found to have mBCC with osseous metastases. To our knowledge, this is the first report of mBCC in the orthopaedic literature. Orthopaedic oncologists should consider mBCC in patients diagnosed with carcinoma of unknown origin, with a known history of BCC, or individuals with light skin pigmentation and age 50 or greater. This can help clinicians make the correct diagnosis and provide the appropriate treatment.

## 1. Introduction

Cutaneous basal cell carcinoma (BCC) is the most commonly diagnosed skin cancer, constituting ~80% of nonmelanoma skin cancers and with incidence rising [1, 2]. Rates are highest in elderly white men, and identified risk factors include UV exposure, fair complexion, immunosuppression, and ionizing radiation or arsenic exposure. While most clinicians consider it an indolent and at most locally invasive malignancy, it can in fact metastasize through both lymphatic and vascular means. Incidences of metastasis are estimated at 0.0028% to 0.55% [3, 4].

A recent literature review spanning 1981-2011 identified only 172 cases of primary cutaneous BCC with pathology-confirmed metastasis not resulting from direct tumor spread [5]. When these results are added to the preceding literature, only ~400 cases of metastatic BCC have been reported [5, 6]. Analysis of 100 patients from the 1981-2011 cohort with adequate follow-up showed patients with distant metastases were diagnosed at a younger age (mean 58.0 years) than those with regional metastases (mean 66.3 years) and had shorter interval survival (24 vs. 87 months). Of the 24 identified patients with metastasis to bone, 20 had vertebral lesions and 9 had rib lesions. The patients with bone metastases had median survival of 12 months, significantly shorter than the 26-month mean without bone metastases. Fourteen of the 24 also had metastases to multiple sites [7]. Among those with mBCC, lymphatic spread may predict longer survival times than hematogenous spread [8].

We report here on a patient recently treated at our institution who was found to have cutaneous BCC metastatic to multiple bony sites, including her ilium, sacrum, inferior public ramus, mandible, occipital condyle, ribs, and lumbar spine. To our knowledge, this is the first report of BCC with bony metastasis to be published in the orthopaedic literature.

## 2. Case Report

The patient, a 71-year-old Caucasian female with prolonged smoking history and inconsistent medical care, presented to our institution via EMS following a mechanical fall she attributed to her chronic right hip pain. She had last been seen in our health system 1.5 years before and had intermittently received care in multiple area healthcare systems. She had known COPD with active smoking, periodontal disease, sick sinus syndrome status postpermanent pacemaker implantation, and urinary frequency. During her admission she was also diagnosed with type 2 diabetes mellitus and congestive heart failure. She had no history of industrial exposures. Her only medications included albuterol and occasional inhaled corticosteroids.

**Figure 1:**
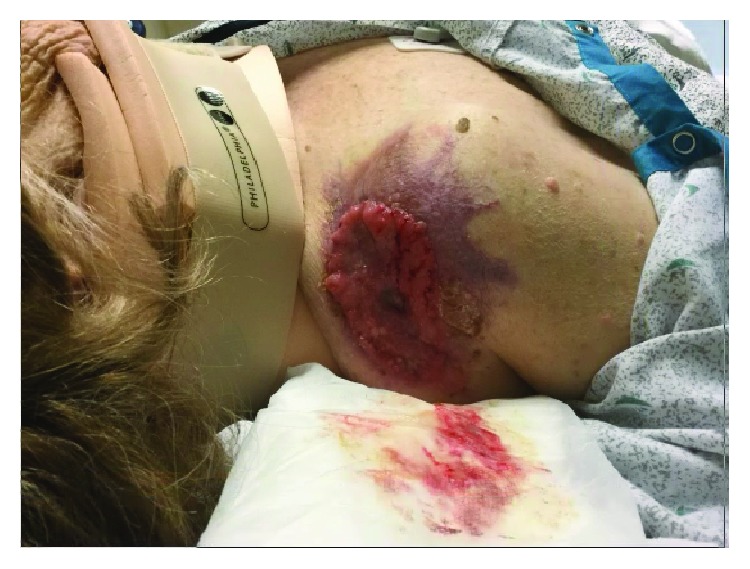
Clinical image of the patient's fungating right shoulder mass, noted on presentation and measuring 6.1 × 5.5 cm. This had reportedly been present many years, with 2 years of noted increasing size, drainage, and friability.

In the ED, workup revealed a large (6.1 × 5.5 cm) fungating right shoulder mass (Figure 1), and the X-ray of her pelvis showed a pathologic fracture of the right iliac wing. She underwent CT of her pelvis, spine, and right shoulder without contrast, of her head with and without contrast, and of her chest/abdomen/pelvis with PO and IV contrast. These studies were remarkable for a pathologic fracture of the right iliac wing, multiple additional pelvic lytic lesions including the sacrum, right inferior pubic ramus, and left iliac wing, and lytic lesions in the right mandibular condyle, left occipital condyle, left posterior rib, right third rib, and L2 vertebral body, all concerning for metastatic disease. The shoulder CT suggested the overlying mass did not extend into underlying muscle but did identify several subcutaneous satellite nodules. The CT abdomen/pelvis showed left renal parenchymal irregularity suspicious for malignancy and bilateral adrenal nodules concerning for metastases.

On questioning, the patient reported she had fallen due to worsening of her chronic right hip pain. Her daughter reported that her right shoulder lesion had been draining, bleeding, and enlarging for two years but had been present for many years longer and that her mother had been told repeatedly to see a dermatologist but had never followed up.

**Figure 2:**
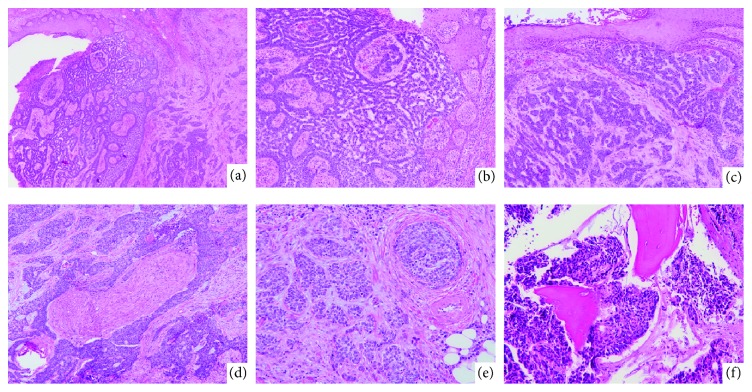
Microscopic findings of the right shoulder mass (a-e) and right iliac crest metastasis (f). (a) Low power image shows ulcerated BCC adjacent to normal skin. The tumor consists of large nodules and irregular angulated nests of tumor cells with peripheral palisading and surrounding myxoid stroma infiltrating into the deep dermis (*hematoxylin-eosin, original magnification 40x*). (b) Some areas of the BCC show histologic pattern of nodular subtype (*hematoxylin-eosin, original magnification 100x*). (c) Other areas of BCC show histologic pattern of morpheaform subtype (*hematoxylin-eosin, original magnification 100x*). (d) Foci of perineural invasion by BCC in the deep dermis (*hematoxylin-eosin, original magnification 100x*). (e) Foci of lymphovascular invasion by BCC (*hematoxylin-eosin, original magnification 200x*). (f) The right iliac crest biopsy shows metastatic BCC wrapping around bone trabeculae with morphological features similar to the right shoulder mass (*hematoxylin-eosin, original magnification 200x*).

She was seen by dermatology in the ED, who performed a shave biopsy of her right shoulder mass on hospital day (HD) 1. She was admitted to a medical service with orthopaedic oncology and medical oncology following. Medical oncology felt her most likely diagnosis was renal cell carcinoma but proposed that multiple myeloma or metastatic skin cancer also needed consideration. The pathology from her initial shave biopsy returned as nodular, ulcerated basal cell carcinoma extending to the base of the sections. Because of the small size of her adrenal masses and the inaccessibility of her renal mass, she underwent fine needle aspiration and biopsy of her right iliac crest bony lesion on HD2. The pathology for this was reported as “metastatic carcinoma morphologically consistent with patient's known basal cell carcinoma of the skin” (Figure 2(f)). Immunohistochemical staining showed the tumor cells were positive for p40, CK 5/6, focally positive for p63, BerEP4, GATA-3, and negative for CK 7, CK 20, CDX2, PAX-8, TTF-1, napsin, and uroplakin II, supporting the diagnosis [9].

On HD6, due to the fungating nature of her right shoulder lesion and persistent bleeding, she underwent wide local excision of her shoulder mass. This pathology again showed ulcerated basal cell carcinoma with features of nodular and morpheaform subtypes invading to a depth of 1.0 cm, with negative margins. There was evidence of perineural invasion and one focus suspicious for lymphovascular invasion (Figures 2(a)–2(e)).

**Figure 3:**
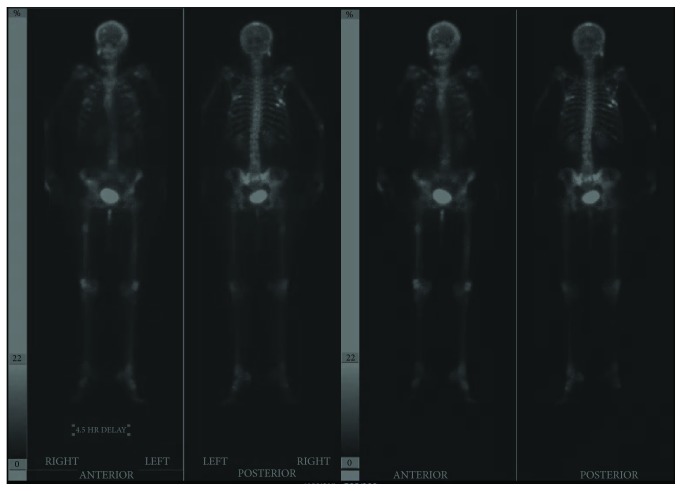
Images from a Tc-99m-MDP bone scan showing multiple focal areas of increased tracer uptake consistent with metastases in the skull, right zygomatic arch, anterior and posterior ribs, right scapula and clavicle, lumbar spine, pelvis, and bilateral femoral shafts.

In addition to the previously imaged osseous lesions, a technetium bone scan performed on HD4 demonstrated metastatic lesions in the right and left mid femoral shafts (Figure 3). CT of the right femur without contrast performed on HD9 showed a 4.2 cm lytic metastatic lesion in the posterolateral middiaphysis with associated cortical thinning and endosteal scalloping, involving two-thirds of the cortical thickness. She underwent prophylactic cephalomedullary nailing of her right femur on HD13. She was treated by radiation oncology with 30 gray in 10 fractions to her right hemiplevis and 8 gray to the right femur postoperatively.

Additionally, she underwent biopsy of a suspicious right leg skin lesion on HD8. The pathology was consistent with melanoma.

On discharge from the hospital, the patient followed up with oncology and was schedule for a PET/CT to better assess her adrenal lesions and for wide reexcision of her right leg melanoma. She was to begin a hedgehog inhibitor (vismodegib) after her PET/CT. However, she was lost to follow-up and represented to our ED 7 weeks after discharge with shortness of breath. She was found to be in diastolic heart failure and to have a subsegmental pulmonary embolus and was started on therapeutic low molecular weight heparin. She was also found to have a new right scapula pathologic fracture. She was discharged to inpatient rehabilitation and expired 3 weeks later.

## 3. Discussion

Here, we have described a case of cutaneous BCC with bony metastasis. To our knowledge, this is the first that such case was described in the orthopaedic literature. Most orthopaedic clinicians are likely not aware that BCC can metastasize to the bone and therefore may not include it on the differential of a metastatic bone lesion, even in a patient with known prior cutaneous BCC. The same can be said of most pathologists, who may not choose stains to identify metastatic cutaneous BCC.

Weissferdt et al. recently reported on the clinicopathological and immunohistochemical findings in 15 cases of mBCC [9]. They suggest that distant metastasis from BCC should be included on the differential for any basaloid carcinoma of the thorax or bone. All but 1 of their patients had a previously diagnosed BCC at the time metastasis was identified. Most (*n* = 11) of their patients had metastasis to the lung, with one to the heart and 3 to the bone (*n* = 1 to femur, *n* = 1 to iliac crest, and *n* = 1 to sacrum). One patient with initial lung metastases later developed bone metastasis as well. Eight of their patients died at a mean of 27 months from mBCC diagnosis, while 7 remained alive at a mean of 29 months after diagnosis. Histologically, 5 were classified as nodular BCC, 3 as basosquamous, 3 mixed, and 1 each morpheaform, micronodular, infiltrative, and superficial. Initially, 8 cases were diagnosed at mBCC, 5 as primary nonsmall cell lung cancer, and 1 as metastatic carcinoma of unknown origin. Two of the bone metastases were initially diagnosed as mBCC and 1 as metastatic carcinoma of unknown origin. Regarding immunohistochemistry, 7/10 cases were positive for bcl-2, 9/11 for BerEP4, and 10/10 negative for EMA (a marker commonly found in SCC) [10–12]. The authors point out that of the primary bone lesions, adamantinoma is histologically similar to mBCC. Clinically, adamantinomas tend to occur in younger patients (generally in their teens and 20s, though they have been reported in 5 year olds to 79 year olds), most commonly in the tibial diaphysis. On radiographs, they appear as expansile lytic lesions commonly with concurrent fibular involvement, and on MRI, they are T1 hypointense and T2 hyperintense lytic intramedullary lesions with cortical involvement [13, 14]. Histologically, they appear less cytologically aggressive and may stain positive for keratin, CK1, CK5, CK14, vimentin, TGF beta, and EMA [13, 15, 16].

The management of localized BCC is typically straightforward, with most lesions amenable to treatment with cryotherapy, curettage and electrodesiccation, marginal excision, or Mohs micrographic surgery. However, the treatment of locally advanced or metastatic BCC is complex. Historically, patients with more advanced lesions received platinum-based agents with reported favorable responses in 38% of patients [7]. More recent targeted therapy has been developed based on the knowledge that improper signaling within the Hedgehog pathway is present in and contributes to the pathogenesis of a large percentage of localized and metastatic BCCs [17, 18]. Two such Hedgehog pathway inhibitors, vismodegib and sonidegib, have shown promising results in the treatment of locally advanced and metastatic BCC and are now approved by the Food and Drug Administration (FDA) and European Medicines Agency (EMA) [19, 20]. A recent meta-analysis [21] of eight studies and 704 patients was performed that demonstrated a response range of 28.0% to 100% and a weighted average of 64.7% (95% CI, 63.7%-65.6%) of patients with locally aggressive BCC to vismodegib. The response range of patients with mBCC was 30.8% to 38.0% with a weighted average of 33.6% (95% CI, 33.1%-34.2%) [21]. Adverse events have been reported in 30% of patients and include muscle spasms, weight loss, fatigue, alopecia, and dysgeusia [19]. Results for sonidegib have been similarly promising for locally advanced BCC with a response rate of 57.5%, but less promising for mBCC, with a response rate of only 17% [22]. Additionally, basic science suggests vismodegib may sensitize BCC to radiation therapy [23]. Additional targeted Hedgehog pathway therapies are being investigated, as early evidence suggests resistance to vismodegib and sonidegib, both inhibitors of the smoothened (SMO) protein, often develops through SMO mutation or downstream Hedgehog pathway alterations [24].

In conclusion, while rare, metastatic BCC should be considered in the differential for bone lesions in patients with known history of BCC. In patients where histology shows carcinoma of unknown primary, especially with basaloid appearance on histology or in Caucasian patients in their 50s or later, the orthopaedic oncologist should specifically assess for suspicious skin lesions or any known history of cutaneous BCC. Inclusion of this diagnosis on the differential could allow pathologists and medical oncologists to better diagnose and treat these patients, improving morbidity and mortality.
